# Analyzing the Elephant: The Search for Electroencephalography Biomarkers

**DOI:** 10.1016/j.bpsgos.2025.100459

**Published:** 2025-05-15

**Authors:** Sydney E. Smith

**Affiliations:** Neurosciences Graduate Program, University of California, San Diego, La Jolla, California

Thousands of years ago, a group of blind men came across a large, strange object: an elephant. None of the men had ever encountered an elephant before, so they decided to use their sense of touch to identify what it might be. After touching the creature’s trunk, one of the men declared the object a snake. Another man placed his hand on its flank and concluded that it was a wall. Yet another man felt the elephant’s tail and decided that it must be a rope. Of course, no one blind man was right—each individual measurement was incomplete and thus provided a misleading picture of the object’s identity. Among its many lessons, this ancient parable tells us that our perception of truth is highly dependent on our limited observations. Analyzing the brain’s electrical signals can be quite similar, but instead of touch, we use signal processing methods to describe the brain’s electrical activity. Interpreting these analyses can be much like the blind men relying on their tactile observations of the elephant, i.e., incomplete and subject to the limitations of our measurements.

SEE CORRESPONDING ARTICLE NO. 100435

Neural signal processing is relevant to psychiatry in the search for biological indicators—or biomarkers—for common and debilitating mood disorders such as depression. With a demand for new depression interventions comes a demand for biomarkers that can aid diagnosis, streamline precision treatment, and illuminate the neural mechanisms of novel therapeutics. Electroencephalography (EEG) is an ideal source of biomarkers because it is cost-effective, mobile, and noninvasive. However, EEG is also a noisy measurement, and the signals that we record from an electrode on the scalp represent the sum of countless, simultaneous processes being carried out by populations of neurons in the brain (1). It is challenging, if not impossible, to determine precisely the physiological drivers of any given change in EEG activity, because many different neural sources can lead to the same quantitative observation.

**Figure 1:**
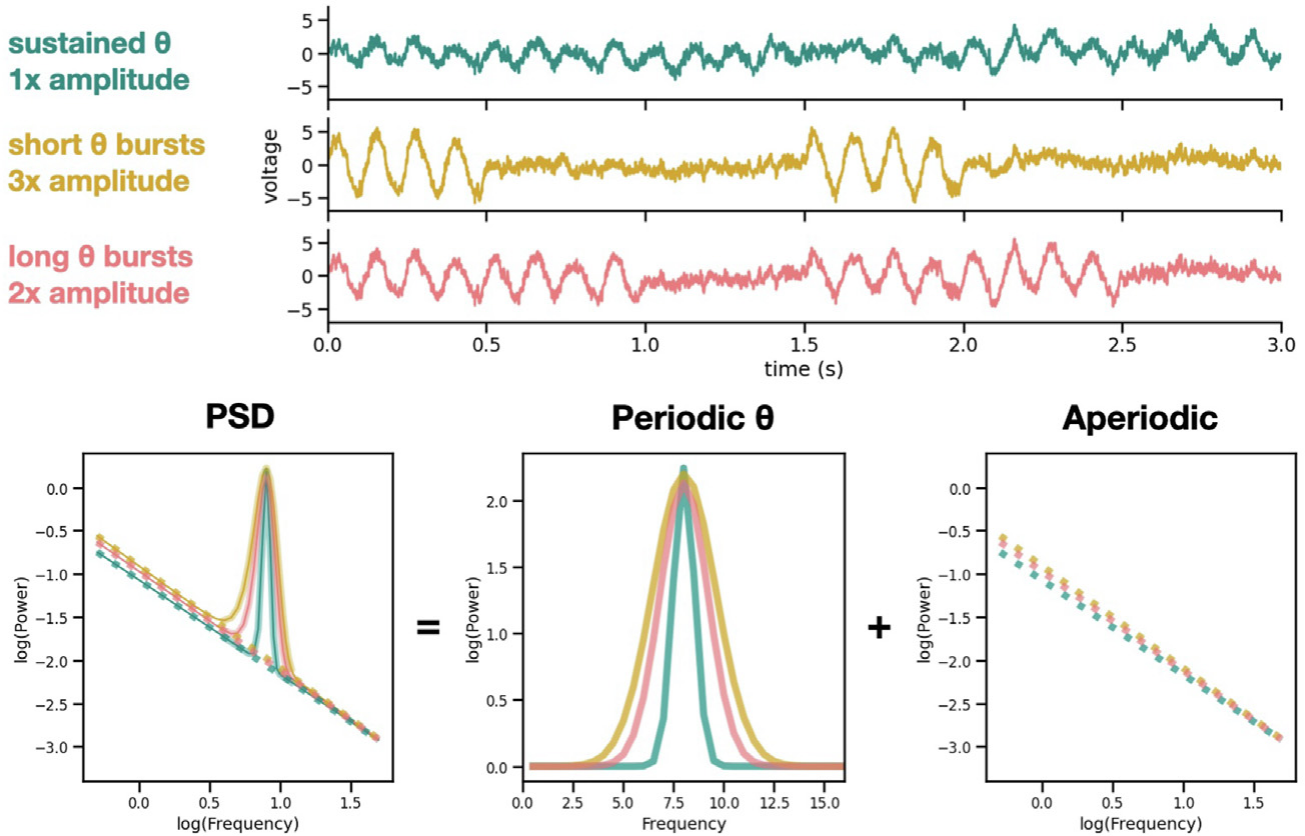
Signals with different characteristics can produce similar analytic outputs in power spectra. Top: Three neural signals are simulated, each consisting of aperiodic activity and a theta (8 Hz) oscillation. The first signal (teal) contains a sustained theta oscillation that lasts for the entirety of the signal. The second signal (gold) contains a bursty, high-amplitude theta oscillation. The amplitude of the bursting theta oscillation is 3 times the amplitude of the sustained theta oscillation above it. The third signal (coral) contains a bursty, medium-amplitude theta oscillation. Its bursts are twice as long as those of the second signal, and the amplitude of its theta bursts are twice the amplitude of the sustained theta (teal) oscillation. Bottom: The PSDs of the simulated signals. The PSDs can be decomposed into their periodic and aperiodic components. Despite each signal having such different characteristics in the time domain, in the PSD, the power of all 3 signals at 8 Hz is equal. PSD, power spectral density.

For example, consider a neural signal that contains a theta oscillation, which is a rhythmic fluctuation in voltage around 8 Hz (Figure 1, top). Theta oscillations are nearly ubiquitous in hippocampal EEG signals recorded from rats, often occurring in bursts that last up to a few seconds and that are associated with spatial navigation and memory. Neural activity, such as a signal that contains a theta oscillation, can be quantified and analyzed using neural power spectra. The power spectrum, or power spectral density (PSD), of an EEG signal is computed by calculating the power of neural activity across many frequencies, with neural activity (or power; y-axis) plotted against frequency (hertz; x-axis). In PSD, a theta oscillation can be seen as a bump of exponentially higher power concentrated at 8 Hz that rises above the sloping, background aperiodic activity (Figure 1, bottom) (2). Although the exact location of this bump on the x-axis can vary by a few hertz, the height of the theta bump above the aperiodic activity is an estimation of how much theta is in the signal. However, this is where our methods can mislead us, just like the blind men touching the elephant.

To illustrate this, I will demonstrate how several different changes in a neural signal can lead to the same output in its PSD. In Figure 1, I have simulated 3 signals with different properties. These signals all contain theta oscillations and aperiodic activity, and they have very similar PSDs, including theta bumps with identical power at 8 Hz (Figure 1, bottom-middle). However, the signals themselves are visibly different. In the first signal, the theta oscillation is long and sustained; the second signal contains short, high-amplitude bursts of theta; and the last signal has slightly longer, medium-amplitude bursts. If you only looked at theta power at exactly 8 Hz, you would not be able to distinguish between these 3 signals and therefore would miss details that are so clearly different when the signal is plotted as voltage that fluctuates over time. These details are important because the generation—and disruption—of oscillations is directly related to the coordinated, time-dependent firing of different types of neurons (3).

But what does this have to do with biomarkers in psychiatry? Clinically useful biomarkers need to be specific and interpretable. For use in diagnosis and as predictive indicators for treatment, they need to be unique. If many different signals can lead to the same quantitative measurement (as in Figure 1), that measurement’s power as a biomarker is diminished. In addition, if a biomarker is being used to understand a therapeutic mechanism at the level of neural circuits, it should reflect the physiological sources of the neural activity as accurately as possible. These sources are inherently dynamic, and their changes over time can directly reflect properties of the neural circuitry that produces them. Therefore, if a neural signal is being used as a biomarker, it should be thoroughly inspected in both the PSD and in the time series. This is precisely what Mazza *et al.* (4) in the previous issue of *Biological Psychiatry: Global Open Science* have accomplished in their search for a biomarker of neural inhibition in the treatment of depression.

Neural inhibition has garnered increasing attention in depression research. Specifically, reduced inhibitory activity has been linked to depression pathology (5). Highly effective stimulation treatments like electroconvulsive therapy have been associated with increases in inhibition using EEG (6), but electroconvulsive therapy’s side effects make it unfavorable for most patients with depression. To induce a similar increase in inhibition without the negative effects, new medications are being developed that precisely target inhibitory cell types in the cortex. These medications can be contrasted with nonspecific modulation by existing medications such as benzodiazepines, which increase inhibition but have undesirable side effects. Reliable EEG biomarkers for these new inhibition-boosting compounds would supplement their further development and eventual clinical use. An EEG biomarker could enable researchers to understand a medication’s effects on neural circuits and/or allow clinicians to monitor how it changes brain activity and precisely target dosages to maximize therapeutic potential.

The search for such a biomarker led Mazza *et al.* (4) to use EEG to monitor the effects of a new inhibition-boosting ligand, an α5-GABA_A_ (gamma-aminobutyric acid A) subunit positive allosteric modulator referred to as α5-PAM. α5-PAM was recently developed as a novel pharmacological intervention for depression, and 2 co-authors are listed as inventors on the associated patent. Identifying clinically relevant EEG signatures of α5-PAM in animal models could aid its implementation in human patients, with EEG as a cost-effective biomarker of the medication’s effects. In their study, 10 rats were injected with α5-PAM at 3 different doses. Their EEGs were compared to the effects of another inhibition-inducing drug, diazepam, and to an injection of vehicle (an inert substance, such as a placebo) as a control condition.

Accounting for aperiodic activity in EEG power spectra, α5-PAM decreased spectral power in the theta bump and decreased its center frequency (the bump’s location on the PSD’s x-axis). But how did the theta oscillation in the neural signal actually change to drive these effects seen in the PSD? To investigate this, Mazza *et al.* (4) looked at the signal in the time domain. By using a method that measures theta oscillations on a cycle-by-cycle basis (7), they investigated what changes in the neural signal (theta amplitude, period, burstiness, etc.) might be driving the observed change in the PSD. They found that α5-PAM increased the theta period (slowing it down), and this was expected from the decrease in theta center frequency observed in the PSD. The decrease in theta power seen in α5-PAM was driven by a decrease in theta burstiness, but the amplitude of individual cycles in the theta oscillation was unchanged compared with baseline and vehicle. Together, these observations indicate that α5-PAM does not change the amplitude of theta bursts; it just makes bursts occur less often than at baseline. This decrease in theta bursts may be related to α5-PAM’s upregulation of a specific type of inhibitory neurons found in the frontal cortex and hippocampus, which have previously been linked to both theta oscillations and depression. The authors would not have discovered this if they had not extended their analysis beyond the PSD.

The EEG signatures of α5-PAM exhibited a dosage response, and its PSD and time domain properties were unique from those of rats administered diazepam or vehicle. By analyzing both the PSD and the time domain features of theta oscillations in EEG, Mazza *et al.* (4) identified distinct biomarkers of α5-PAM response in rats. In the future, these biomarkers can be used to interrogate α5-PAM effects on the level of neural circuits, and they have the potential to be extended to look for analogous EEG signatures in humans in the clinic. By combining neural PSD analysis with an examination of the signal in the time domain, the authors’ approach to establishing a viable biomarker of α5-PAM response avoided pitfalls of signal analysis that could have misled them. Unlike the blind men whose incomplete observations lead them to mistaken conclusions in the parable of the elephant, Mazza *et al.* (4) have gotten closer to establishing a useful clinical biomarker for a promising new medication for depression.
